# Sensory Improvement of a Pea Protein-Based Product Using Microbial Co-Cultures of Lactic Acid Bacteria and Yeasts

**DOI:** 10.3390/foods9030349

**Published:** 2020-03-17

**Authors:** Cynthia El Youssef, Pascal Bonnarme, Sébastien Fraud, Anne-Claire Péron, Sandra Helinck, Sophie Landaud

**Affiliations:** 1 General Mills Yoplait, Vienne Technical Center, 38205 Vienne, France; Cynthia.Elyoussef@genmills.com (C.E.Y.); Sebastien.Fraud@genmills.com (S.F.); 2 Université Paris-Saclay, INRAE, AgroParisTech, UMR SayFood, 78850 Thiverval-Grignon, France; pascal.bonnarme@inrae.fr (P.B.); anne-claire.peron@inrae.fr (A.-C.P.); sophie.landaud@agroparistech.fr (S.L.)

**Keywords:** pea protein, fermentation, lactic acid bacteria, yeast, beany, green

## Abstract

Consumer demands for plant-based products have increased in recent years. However, their consumption is still limited due to the presence of off-flavor compounds, primarily beany and green notes, which are mainly associated with the presence of aldehydes, ketones, furans, and alcohols. To overcome this problem, fermentation is used as a lever to reduce off-flavors. A starter culture of lactic acid bacteria (LAB) was tested in a 4% pea protein solution with one of the following yeasts: *Kluyveromyces lactis*, *Kluyveromyces marxianus*, or *Torulaspora delbrueckii*. The fermented samples were evaluated by a sensory panel. Non-fermented and fermented matrices were analyzed by gas chromatography coupled with mass spectrometry to identify and quantify the volatile compounds. The sensory evaluation showed a significant reduction in the green/leguminous attributes of pea proteins and the generation of new descriptors in the presence of yeasts. Compared to the non-fermented matrix, fermentations with LAB or LAB and yeasts led to the degradation of many off-flavor compounds. Moreover, the presence of yeasts triggered the generation of esters. Thus, fermentation by a co-culture of LAB and yeasts can be used as a powerful tool for the improvement of the sensory perception of a pea protein-based product.

## 1. Introduction 

The demand for plant-based products is on the rise as consumers become increasingly health-conscious and seek out non-dairy alternatives for dairy products. Reasons for the plant-based movement stem from three different factors: environmental, health, and concerns about animal welfare [1,2]. Consequently, the food industries are undoubtedly experiencing disruptive pressures in favor of plant-based products and are evolving rapidly to meet consumer needs [3].

Due to their high protein content ranging from 20% to 25%, pulses present an important economic and nutritional interest [4]. These dried seeds belong to the family of legumes, Fabaceae, which includes *Pisum sativum* (peas), *Cicer arietinum* (chickpeas), *Lupinus* (lupins), *Phaseolus vulgaris* (beans), *Glycine* max (soybeans), and *Arachis hypogaea* (peanuts) [5]. As a result of their limited amount of sulfur-containing amino acids (methionine, cystine, and tryptophan), they are often consumed along with cereal grains that have a complementary amino acid profile, i.e., that are rich in sulfur-containing amino acids but deficient in lysine. In addition to their high nutritive value, the consumption of pulses can offer many health benefits to human beings, e.g., the reduction of cardiovascular diseases, diabetes, and cancer risk [6,7]. 

Thanks to its functional properties and high protein content, pea (*Pisum sativum*) offers a great opportunity to develop an alternative plant protein source that will meet nutritional needs in an affordable and sustainable way [8]. This plant-based protein can be used in food applications as an isolate (protein content higher than 80%) or in concentrate form (protein content lower than 80%). However, the presence of off-flavors described as “beany,” “green,” “vegetal,” “hay-like”, and “rancid” is a limiting factor for its consumption [9,10]. These undesirable notes are partially inherent in peas or produced during harvesting, processing, and storage. 

The typical beany flavor of pea is generally associated with aldehyde molecules, mainly hexanal, which is the most frequently reported compound responsible for the undesirable aroma of pea proteins. In addition to aldehyde molecules, other compounds such as ketones, alcohols, pyrazines, and furans also contribute to the off-flavor profile of pea proteins [5]. Thus, the perception of the beany flavor could arise from the interactions between different molecules and would, therefore, not be the result of the perception of one single compound [11]. 

Fermentation, one of the oldest stabilization processes, has been used for centuries as a method to preserve agricultural raw materials and to improve their organoleptic properties. In that respect, the preparation of many traditional cereal- and pulse-based food products relies on the use of fermentation [12]. The diversity of fermented foods depends on the types of substrates and microorganisms involved in the process. Some examples of cereal-based fermented products are beer, bread, and a less common product, boza [13]. Alcoholic beverages are made from different substrates such as malted barley in Western countries (in the case of beer), as well as from sorghum (burukutu) and maize/sorghum (pito) in Africa. These alcoholic beverages are mainly produced using yeasts (*Saccharomyces* sp.) but also use co-cultures of yeasts and lactic and acetic acid bacteria, e.g., *Brettanomyces*, *Saccharomyces*, *Lactobacillus, Leuconostoc,* and *Acetobacter* for lambic beers; *Geotrichum candidum* and *Lactobacillus* for pito; and *S. cerevisae, S. chavelieri, Leuconostoc mesenteroides, Candida,* and *Acetobacter* for burukutu [13,14]. On the other hand, boza, a beverage made from wheat, rye, millet, maize, and other cereals, is mainly fermented with lactic acid bacteria (LAB) such as *Lactobacillus* and *Leuconostoc* sp., and sometimes associated with yeasts (*S. cerevisiae*) [13]. Bread, which is also known as naan, bhatura, and kulcha in Asian countries, is a dough obtained mainly by yeast fermentation (*S. cerevisiae*) but can also be produced via a co-culture of LAB and yeasts [15]. 

In addition to fermented cereal-based beverages, fermented food products made from pulses are generally found in traditional Asian food products. One of the major types of pulses used is soybean, which contributes to the formation of a wide range of products such as sufu, tempeh, miso, and natto [16]. As in the case of cereal-based beverages, these fermented products can be made with an association of LAB (*Pediococcus*), *Bacillus*, fungi (*Rhizopus, Actinomucor,* and *Mucor*), and yeasts (*Saccharomyce*s*, Candida*) [17,18,19]. It should be noted that several species used in these fermented products, such as *Rhizopus* spp., *Mucor* spp., and *Candida* spp., are not on the Qualified Presumption of Safety (QPS) list or are not recommended for QPS status in European countries [20]. 

Consequently, most plant-based fermented foods are produced using microbial communities due to their potential synergistic effects that help to improve the qualities of fermented products [21]. However, the use of fermentation for pea-based matrices using microbial communities with variable complexities is very rare. 

Previous studies have reported the impact of lactic acid fermentation on the modification of the green/beany perception of pea. They showed that lactic acid fermentation had a limited effect on the reduction of the negative descriptors associated with pea proteins [10,22,23]. Moreover, the addition of yeasts such as *Candida catenulate* and *Geotrichum candidum* triggered the formation of banana and apricot aroma in a cheese-like pea-based product [24].

Thus, a promising line of research would be to co-ferment pea protein with LAB and yeasts. The reason that these microorganisms were combined in our study is based on the need to use LAB to obtain a pea protein “yogurt-like” product with a final pH value of 4.55 that ensures both its sanitary quality and gelation properties. Moreover, the genera *Saccharomyces* and *Kluyveromyces* display enzymatic activities such as alcohol and aldehyde dehydrogenase, enzymes that have been reported to decrease green beany flavor in soybeans through the degradation of aldehydes and alcohols [25]. Yeasts are known to improve the aroma quality of fermented beverages via the formation of ester compounds with fruity and floral notes such as ethyl acetate and 2-methylbutyl acetate [26]. The aim of this study is, therefore, to investigate the impact of fermentation with yeasts in co-culture with LAB on the volatile profile associated with off-flavor in peas. 

The specific objectives of this study are: (1) to evaluate the impact of the addition of yeasts on the acidifying activity of LAB; and (2) to compare the flavor profile of the samples fermented with a pure culture of LAB and the ones fermented with LAB in co-culture with yeasts. 

## 2. Materials and Methods

### 2.1. Raw Materials, Ingredients, and Strains

Pea protein isolates of *Pisum sativum L.* (Purispea Tm 870, batch1708TL1) were supplied by Cargill (Chicago, IL, USA). The sample had the following characteristics: pH 7.0, moisture 4.2%, and protein content 82.1%. Sucrose from sugar cane was provided by Tereos (France). The raw materials were stored at room temperature.

VEGE 047 LYO was obtained from DuPont Danisco (Dangé-Saint-Romain, France) and consisted of freeze-dried defined strains of lactic acid bacteria: *Lactobacillus acidophilus* NCFM^®^*, Streptococcus thermophilus*, *Lactobacillus delbrueckii subsp. bulgaricus*, and *Bifidobacterium lactis* HN019™. Using API 50 CHL medium (Biomérieux SA; Marcy l’Etoile, France), this mixture of lactic acid bacteria and *Bifidobacterium* was found to be glucose (+), fructose (+), and sucrose (+). *Torulaspora delbrueckii* TD 291 (freeze-dried BIODIVA^TM^) was provided by LALLEMAND S.A.S, France. *Kluyveromyces lactis* Clib 196 and *Kluyveromyces marxianus* 3810 were obtained from the INRA collection (UMR GMPA, Grignon, France).

### 2.2. Fermentation of Pea Protein Isolates

#### 2.2.1. Inoculum Preparation

*Kluyveromyces lactis* Clib 196 and *Kluyveromyces marxianus* 3810 were incubated from frozen glycerol stocks (−80 °C) in potato dextrose broth (PDB, Becton, Dickinson & Company, Sparks, MD, USA) for 21 h at 30 °C with an agitation of 200 rpm using an incubator (INFORS HT). When the stationary phase of growth was reached, cells were harvested by centrifugation at 4000 rpm (1699× *g*) for 15 min at 10 °C (Eppendorf, 5804R). The pellet was then washed and suspended in sterile physiological water (9 g of NaCl in 1 L of osmotic water) to obtain 10^7^ colony forming unit/mL (CFU/mL) and used as the inoculum.

For the rehydration of VEGE 047 LYO, we followed the supplier’s instructions. The freeze-dried culture was rehydrated in a 500-mL of a 4% thermally treated pea protein solution using Purispea Tm 870. The mixture was left to settle for 15 min and then agitated gently before the preparation of the cryotubes. The latter was then stored at −80 °C.

As for *Torulaspora delbrueckii*, 0.25 g/L was inoculated in a pea protein solution and then transferred to cryotubes at −80 °C until the fermentation process.

Upon fermentation, the samples were inoculated with 10^7^ CFU/mL of VEGE and *Torulaspora delbrueckii*.

#### 2.2.2. Preparation of Fermented Pea Protein Isolate

Osmotic water was used to prepare a 4% pea protein solution with 3% sucrose. The mixture was stirred using a magnetic stirrer at room temperature for 10 min until a homogeneous solution was obtained. The solution was then thermally treated at 110 °C for 15 min. This process was required to eliminate the endogenous microflora of pea proteins before fermentation. Prior to inoculation, the solution was cooled down to 30 °C. The initial pH of the solution was 7.1 ± 0.1.

Four different fermented samples were inoculated with the following strains: VEGE 047 (VEGE), VEGE 047 + *Kluyveromyces lactis* Clib 196 (VEGE + KL), VEGE 047 + *Kluyveromyces marxianus* 3810 (VEGE + KM), and VEGE 047 + *Torulaspora delbrueckii* (VEGE + TOR). The experiments were carried out in triplicate.

Fermentations were stopped at pH 4.55 (an optimal pH to ensure sanitary and textural qualities) by rapid cooling in an ice bath until the temperature reached 4 °C. Microbial enumerations were performed at this point.

All fermented samples were stored in 150-mL glass vials at 4 °C for 7 days. At day 7, the products were sent to an expert panel for sensory evaluation and the remaining samples were frozen at −80 °C until analysis.

### 2.3. Fermentation Monitoring

#### 2.3.1. Acidification Activity Measurement

The Cinac system 4 (AMS, Frépillon France) [27] was used to measure the acidification activity of the microbial strains at 30 °C. The pH of the inoculated pea protein samples was continuously measured and automatically recorded at 3-min intervals. The time to reach pH 4.55 (tpH 4.55 in min) was used as a descriptor for the acidification activity. It was calculated using Cinac 4 (version 4, release 0.4.4). Measurements were made in triplicate.

#### 2.3.2. Microbial analyses

LAB and yeast populations were determined at the beginning and at the end of the fermentation process when a pH of 4.55 was reached. The samples were diluted 1:10 with sterile physiological water (9 g of NaCl/L) and then homogenized using an Ultra Turrax^®^device (Labortechnik, Germany) at 8000 rpm for 1 min. The yeast population was determined by surface plating in triplicate using yeast–glucose–chloramphenicol agar (YGCA, BIOKAR, Beauvais, France) after three days of incubation at 30 °C. Lactic acid bacteria were counted by spread plating technique in triplicate on non-acidified Man Rogosa and Sharpe agar (MRS, BIOKAR, Beauvais, France) after 3 days at 42 °C under anaerobic conditions (Bugbox Anaerobic System, Ruskinn, Bridgend, United Kingdom).

#### 2.3.3. Biochemical Analysis

##### Analyses Using HPLC–MS to Determine Sugar Content

To obtain an accurate determination of the amount of sucrose, fructose, and glucose in a complex matrix, a sugar analysis was done using liquid chromatography coupled with mass spectrometry.

Sugars were extracted as previously described [28]. After thawing, the samples were diluted in 50/50 (*v/v*) LC/MS water/acetonitrile and were quantified using high-performance liquid chromatography coupled with mass spectrometry (Waters, Beaver Dam, WI, USA).

Metabolites were separated on an XBridge BEH Amide column (length: 150 mm; internal diameter: 4.6 mm; particle size: 3.5 µm; WATERS). The column temperature was set at 75 °C. The flow was 0.4 mL/min and the solvent were acetonitrile with 0.1% formic acid (D) and ultra-pure water (B) + 0.1% formic acid. The elution gradient was as follows: 0 min at 80% D + 20% B, then 50% D + 50% B for 23 min, level at 80% D and 20% D for 2 min. The injection volume was 5 µL, and the injector temperature was 7 °C. Each analysis took 25 min.

Mass spectrometric detection was performed with an ISQ™ EC-LC Quadrupole with a heated electrospray source (HESI–II) operated in the negative ionization mode (Thermofisher Scientific). Metabolites were identified and quantified (ng/g wet weight) using Chromeleon 7.2.10 software (Thermofisher scientific, Waltham, MA, USA).

##### Analyses Using HPLC to Determine Ethanol and Lactic Acid Concentrations

The concentrations of ethanol and lactic acid were determined by high performance liquid chromatography (HPLC). Similar to the preparation of sugar extracts, ethanol and lactic acid were extracted as previously described [28].

The analysis was performed using a Waters Associates chromatographic system (Alliance) equipped with a pump, an automatic injector (Waters e2695) and two columns, a pre-column of 30 × 4.6 mm (Bio-Rad Labs; Richmond, CA, USA), and an HPX-87H columns (300 × 7.8 mm; Bio-Rad Labs; Richmond, CA, USA) connected in series. The columns were operated at 35 °C. The samples were eluted with 0.01 N sulfuric acid at a flow rate of 0.6 mL/min. The eluting compounds were detected by a UV detector (Model 2489). This detector was connected in series to an RI detector (Model 2414); Empower ^TM^ 3 chromatography data software (Waters Corporation) was used to integrate peak areas using calibration by an external standard solution.

### 2.4. Sensory Evaluation

The sensory evaluation of the four products was performed using descriptive analysis. A panel of 15 trained panelists was recruited for their familiarity with plant-based products.

Sensory analysis was carried out in an air-conditioned room (20 °C), in individual booths, under daylight. Samples of the fermented products (80 g) were presented in plastic cups labeled with randomly selected three-digit numbers. The sample evaluation order was balanced over the panel following a Williams Latin square design to account for potential order and carry-over effects. Panelists were asked to rinse their mouths with water and crackers between samples.

A one-hour session was dedicated to the generation of attributes, followed by training in the use of these attributes to obtain a quantitative description of the products. The 15 panelists generated a vocabulary of sensory attributes that covered the odor, texture, aroma, and taste of the samples. During the second session, the panelists had to rate the intensity of the 13 attributes (global intensity, sour, bitter, astringent, tangy, sparkling, green flavor/vegetal, leguminous plant, citrus fruit, nut, beer/yeast, sourdough, cultured apple cider) generated for each product on an interval scale ranging from 0 to 15 (from nonexistent to marked). Samples were presented in a monadic sequence. The panel performances were validated in a third session in terms of repeatability, using different means of analysis of variance (ANOVA)

### 2.5. Aroma Compound Analysis

To identify the aroma compounds present in the non-fermented and fermented pea protein solutions, GC/MS analysis was performed. All analyses were performed in triplicate.

Volatile compounds were extracted using the purge and trap method by means of a Gerstel Dynamic Headspace System (DHS) coupled with a Gerstel Multipurpose Sampler (MPS) Autosampler (Mulheim an der Ruhr, Denmark). Five grams of the fermented or non-fermented samples were weighed in a vial. The DHS system heated the samples to 40 °C for 3 min with an agitation speed of 500 rpm. The samples were purged with a helium flow at 30 mL/min for 10 min and analytes (volatile molecules) were collected on sorbent material at 30 °C. The sorbent material used for volatile molecule collection was Tenax TA (2, 6-diphenylene oxide polymer) (Gerstel). The sorbent material was dried to remove residual water vapor at 30 °C with a helium flow of 50 mL/min for 6 min.

GCMS was performed using a 7890 Agilent GC system coupled to an Agilent 5977B quadruple mass spectrometer (Agilent, Santa Clara, CA, USA). A non-polar Agilent column DB-5MS (60 m × 0.32 mm × 1 μm) was used. The injection was performed in splitless mode using helium at a flow rate of 1.6 mL/min. The oven temperature of the column was programmed as follows: temperature increase from 40 to 155 °C at 4 °C/min, followed by 155 to 250 °C at 20 °C/min. The oven temperature was then maintained at 250 °C for 5 min. The gas chromatogram was recorded and analyzed for volatile retention time. Volatile compounds identified by comparison with a mass spectra library (NIST database) were chosen based on their percentage of identity.

For the quantification of the volatile compounds, two different standard solutions were prepared: Solution A containing the molecules responsible for the off-notes, and solution B comprising the ester molecules. The choice of the volatile compounds responsible for the off-flavor perception in pea was based on their occurrence in the literature.

For Solution A, a mixture of 21 selected compounds was prepared. Pure commercial volatile standards belonging to different families of compounds were purchased from Sigma Aldrich (Milwaukee, USA). These standards were as follows: 2-methylpropanal, trans-2-methyl-2-butenal, hexanal, (E)-2- hexenal, heptanal, (E)-2-octenal, nonanal, butanal, (E)-2-heptenal, decanal, 1-penten-3-ol, 1-octen-3-ol, 1-hexanol, 1-octanol, 6-methyl-5-hepten-2-one, 2-octanone, 2-nonanone, 2-n-heptylfuran, 2-ethylfuran, and 2-pentylfuran.

Solution B consisted of ester compounds, major aromatic molecules resulting from yeast fermentation: isoamyl acetate, 2-methylbutyl acetate, 2-phenylethyl acetate, isobutyl acetate, ethyl octanoate, ethyl hexanoate, hexyl acetate, ethyl isobutyrate, ethyl propionate, propyl acetate, and ethyl acetate.

To obtain the calibration curves, four concentrations were prepared for Solution A or B, made from two mother solutions in water and injected three times.

To consider the interactions between aroma compounds and proteins, dilutions were carried out in a 4% sodium caseinate solution (Sigma Aldrich, St. Louis, MO, USA). The choice of the dairy protein is justified by the intrinsic contents of off-flavors in pea proteins, the neutral taste, and the negligible volatile profile of dairy proteins.

### 2.6. Statistical Analysis

One-way analysis of variance (ANOVA) was performed using Xlstat sensory software (version 2019.4.1) (Addinsoft, New York, NY, USA). All tests were performed at *p* = 0.05. The heat map was generated using Euclidean distance and the complete linkage algorithm (90) implemented in the gplots package (version 3.0.1.1) (https://CRAN.Rproject.org/package=gplots) of R software (version 3.6.1) (http://www.r-project.org/).

## 3. Results and Discussion

### 3.1. The Impact of the Addition of Yeasts on Pea Fermentation by LAB

Table 1 summarizes the main parameters that characterize the fermentations performed by the different cultures: the bacterial and yeast biomass at the initial and/or final time (t0 and tf, respectively, where tf is the time necessary to reach pH 4.55), the residual sugar level at tf, and finally the lactic acid and ethanol concentration at tf.

To evaluate the impact of the addition of yeasts on the acidification rate of VEGE, the parameter tf was analyzed. As shown in Table 1, the addition of yeasts to VEGE did not have a significant impact on the fermentation time. This is a positive result in the aim of producing commercial products. However, we can observe a difference in the tf between two yeast species: there was an increase in the time necessary to reach pH 4.55 for VEGE + KM compared to VEGE + TOR. Nevertheless, this cannot be directly linked to the final bacteria biomass since higher concentrations were found in VEGE + TOR.

On the other hand, differences in the final concentration of lactic acid were observed in the presence of yeasts compared to VEGE alone: a lower concentration of lactic acid was observed for VEGE + KM and VEGE + TOR. These differences could be explained by an acidifying metabolite produced by yeasts. In fact, these two co-cultures displayed a higher production of ethanol compared to VEGE alone (in which no production was identified) or in VEGE + KL. Ethanol production paralleled carbon dioxide generation, a gas that, by dissolution, acidifies the medium. Thus, the production of CO_2_ could explain the lower concentration of lactic acid generated to reach pH 4.55 in the two former conditions. As for VEGE + KL, the low final ethanol concentration could explain that the lactic acid concentration is nearly the same in this co-culture compared to VEGE alone. Since *Kluyveromyces lactis* is an aerobic-respiring yeast, it could be limited by the oxygen availability in our static fermentation, leading to limited growth and ethanol production compared to *Kluyveromyces marxianus* [29,30]. 

Considering the sucrose metabolism, the total residual sugars were lower in the presence of yeasts, compared to VEGE. In fact, VEGE alone consumed 6 g/L of sucrose, and no residual monosaccharides (fructose or glucose) were detected (Supplementary Table S1). The yield of lactic acid/sucrose calculated for VEGE was approximately 0.8 g/g, which is close to a homofermentative yield. In fact, VEGE contains three homofermentative species (*Lactobacillus acidophilus*, *Streptococcus thermophilus,* and *Lactobacillus delbrueckii* subsp. *bulgaricus).*

In co-cultures, nearly 100% of the initial sucrose (30 g/L) was hydrolyzed into glucose and fructose, and these monosaccharides were partially metabolized (Supplementary Table S1). The consumed sugars were 9 g/L for (VEGE+KL), 14 g/L for (VEGE + TOR), and 17 g/L for (VEGE + KM). These results are in agreement with the final concentration of ethanol obtained in each condition. As a conclusion, the addition of yeasts had a weak impact on the behavior of VEGE, although slight differences were identified due to probable negative and/or positive interactions between VEGE and the yeast used.

### 3.2. Modification of the Sensory Perception in the Presence of Yeasts

In order to evaluate the impact of yeasts, a sensory analysis was performed only on the fermented products. It was previously shown that VEGE cultures improved the sensory perception of a pea protein yogurt-like product, but not enough for consumer acceptability (General Mills, personal communication).

**Figure 1:**
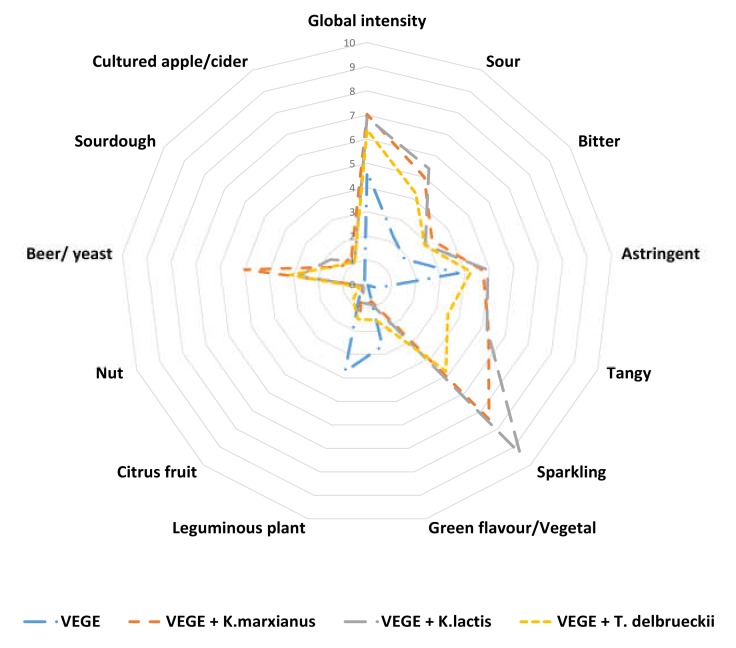
Aroma profile analyses of the four fermented pea protein isolate products. Data are displayed as mean numerical values of the sensory evaluations.

The panelists generated typical descriptors for a plant matrix: green flavor/vegetal, leguminous plant, bitter, astringent, nut, and other descriptors such as sparkling, tangy, sour, citrus fruit, beer/yeast, and cultured apple cider. Figure 1 shows the characteristics of each fermented product as the average intensity of the individual panelists’ scores, and the detailed data are shown in Table 2.

Considering the main defects detected in the pea matrix, the intensities of leguminous plant and green flavor/vegetal were significantly reduced in the presence of yeasts. There is a lack of information in the literature about the impact of yeasts on the improvement of the sensory characteristics of pea proteins. However, one recent study revealed a significant decrease in the beany odor using analytical methods after the fermentation of soybean residue, okara, by *K. marxianus* [31].

The global intensity of aroma in the presence of yeasts was significantly higher compared to VEGE culture. This increase could be linked to the presence of trigeminal sensations such as sparkling, tangy, and sour attributes, which were significantly higher in the samples with yeasts. The sparkling attribute could be directly linked to the presence of CO_2_ [32]. Considering the sour attribute, the yeasts can produce pyruvic and acetic acid, which could explain this perception [33]. In addition, it was previously shown that high levels of carbonation significantly enhanced the sourness and astringency in flavored milk beverages [34].

Finally, the fermented products obtained with yeasts were characterized by a significantly higher “beer/yeast” attribute than the products obtained with VEGE. In fermented beverages such as beer, esters are the most important set of yeast-derived aroma-active compounds, and they are responsible for their fruity character [35].

The modifications of the sensory perception in the presence of yeasts could be attributed to a reduction in the concentration of pea off-notes or the generation of new notes that could modify the perception of sensory defects. Thus, to obtain a better understanding of the sensory modifications, the volatile profiles were analyzed.

### 3.3. Characterization of Volatile Compounds Identified Using GC–MS Analysis

To obtain a better insight into the impact of fermentation on the volatile profile, GC–MS analyses were performed on the uninoculated and fermented samples (VEGE, VEGE + KM, VEGE + KL and VEGE + TOR).

#### 3.3.1. Volatile profiles of Uninoculated and Fermented Samples

**Figure 2:**
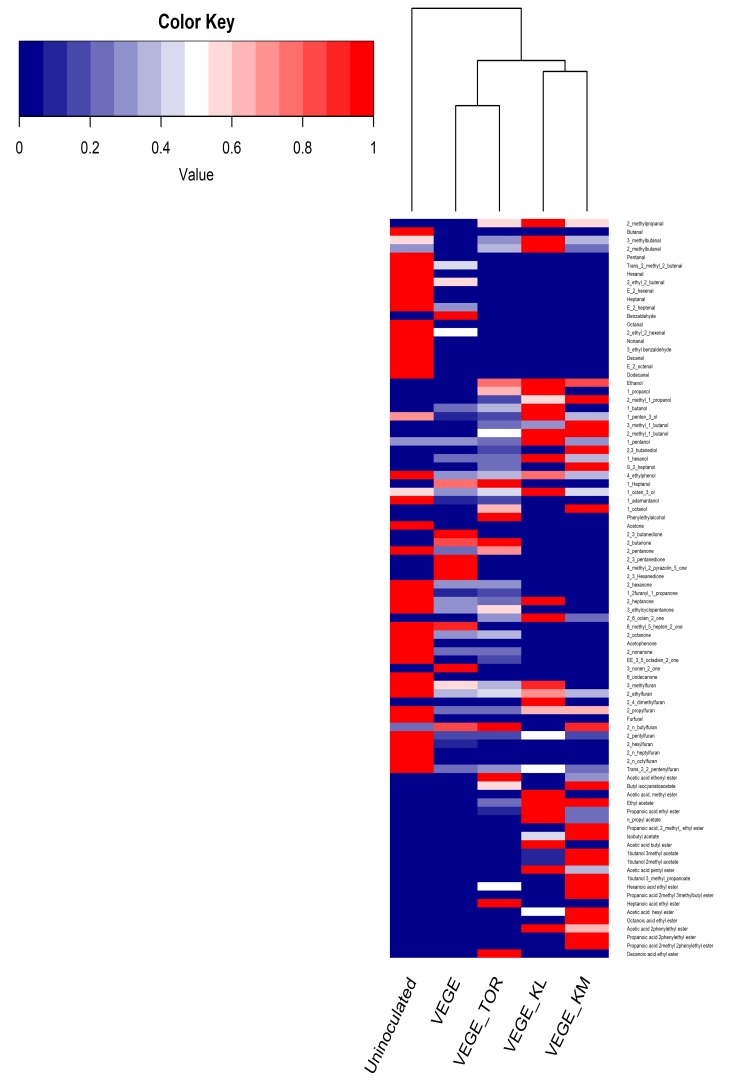
A hierarchically clustered heat map showing the patterns of the different samples for the identified volatile compounds.

A total of 87 volatile molecules were detected. These compounds were grouped into five families, including aldehydes, alcohols, ketones, furans, and esters. A heat map (Figure 2) was drawn up using the proportions of each molecule (proportions calculated using the surface areas of the peaks) among the samples (Supplementary Table S2).

First of all, it was observed that the uninoculated sample contained most of the aldehyde, ketone, and furan compounds, which are major families of molecules responsible for pea off-flavor. Upon fermentation with VEGE or VEGE + yeasts, the volatile profiles were strikingly modified compared to the uninoculated sample. As shown in Figure 2, most of the aldehyde, furan, and ketone molecules were degraded. Moreover, the esters were only present in the fermented products with VEGE and yeasts. The concentrations of alcohol were also generally higher in these products compared to VEGE.

The distribution of the proportions of the molecules revealed the presence of two main groups for the fermented cluster: VEGE and VEGE + TOR vs. VEGE + KL and VEGE + KM. These two clusters were not in agreement with the sensory results, which revealed differences in the sensory perception of products fermented with VEGE or VEGE and yeasts. In fact, there is not a direct link between the volatile compounds identified by GC–MS and the sensory descriptors.

Consequently, to acquire a better understanding of the sensory perceptions, we focused on the off-flavor molecules.

#### 3.3.2. Degradation of Off-Flavor Molecules in the Fermented Samples

Twenty molecules among the 87 aroma compounds detected were reported in the literature as being responsible for the off-flavor perception in peas [9,36,37]. The concentrations of these 20 molecules are presented in Table 3. The perception thresholds were also reported for information purposes because they were determined by orthonasal olfaction in water [38].

First, it should be emphasized that most of the molecules responsible for the pea off-flavors were present in the uninoculated sample in which some of them were detected at high concentrations, including hexanal, butanal, 2-pentylfuran, and 2-ethylfuran. Hexanal has already been reported to be the major molecule responsible for the “green” and “herbal” perception in pea protein isolates [39]. Moreover, hexanal, as well as heptanal and nonanal, were identified as the main volatile compounds of soymilk flavor [40].

In the four fermented samples, there was a significant elimination of off-flavor molecules, mainly aldehydes, ketones, and furans. Previous studies showed that *Lactobacilli* and *Streptococci* could eliminate compounds related to the beany flavor of soymilk during fermentation, such as hexanal and 2-pentylfuran [40,41]. Moreover, lactic acid fermentation with *L. plantarum* or with *P. pentosaceus* had the potential to decrease the concentration of hexanal in lupin protein extracts [22].

Other compounds such as (E)-2-heptenal, 6-methyl-5-hepten-2-one, and trans-2-methyl-2-butenal were present in the products fermented with VEGE, but were not detectable in those fermented with VEGE and yeasts. These differences might explain the fact that the products fermented with VEGE were perceived as being greener and more leguminous by the panelists compared to the products fermented with VEGE and yeasts. This hypothesis should be supported by the determination of the odor perception thresholds by retronasal olfaction in pea.

In the fermented products with VEGE or with VEGE and yeasts, the incomplete reduction or the increase in off-flavor concentrations were notable. For example, 2-pentyl-furan and 2-ethyl-furan were reduced but remained at high levels in all the products. As for 2-methylpropanal, 1-hexanol, and 1-octanol, the concentrations increased when yeasts were added. These compounds could be the result of yeast metabolism under anaerobic conditions. Previous studies showed that the level of 2-methylpropanal increased in fermented pea gels using a microbial consortium, including yeasts [42]. This compound, which contributes to malty and chocolate-like notes, is the result of valine degradation by yeasts through the Ehrlich degradation pathway [43]. As for the two other alcohols, they could have been produced by the reduction of hexanal and octanal through the action of alcohol dehydrogenase activities. It was previously suggested that fermentation of soybean residue by *K. lactis* drastically decreased the amount of hexanal to trace levels, with a corresponding increase in hexanoic acid and/or hexanol [25,26,27,28,29,30,31,32,33,34,35,36,37,38,39,40,41,42,43,44]. The products with yeasts were perceived as being less “leguminous plant and green flavor/vegetal” than the products with VEGE; it is possible that the concentrations of 2-methylpropanal, 1-hexanol, and 1-octanol could be under their perception threshold. As previously mentioned, it will be necessary to determine the odor threshold of the molecules responsible for the sensory defects in peas.

In addition, the perception of the above—mentioned compounds could have been modified by the presence of other molecules generated by yeasts such as esters. It was already suggested that the reduction and/or masking effect of off-flavors led to a more pleasant odor in the fermented lupin protein extracts compared to the unfermented protein extracts [22].

### 3.4. Understanding the Sensory Perception Generated by Yeasts

Esters were only detected in the products fermented with (Supplementary Table S2). Their concentrations, as well as their perception thresholds, are reported in Table 4.

As shown in Table 4, two main groups of esters were identified. The first group includes acetate esters (ethyl acetate, isobutyl acetate, 3-methylbutyl acetate, and 2-phenylethyl acetate), and the second group is composed of the ethyl esters (ethyl hexanoate and ethyl octanoate). The acetate esters in these two groups were found to be produced at higher levels compared to ethyl esters. These esters and their proportions are typical of fermented beverages such as beer [36]. This could explain the beer/yeast descriptor used in sensory analysis, which was shown to be significantly different between products with VEGE and those with VEGE and yeasts.

Ethyl acetate, an undesirable compound, was found to be present in higher concentrations in the three fermented samples compared to the other ester compounds. This molecule was also reported as the most abundant ester in wine and beers [50,51]. However, ethyl acetate could have a limited impact on sensory perception due to its high odor perception threshold [46]. As shown in Table 4, KM and KL produced more ethyl acetate compared to TOR.

Acetate esters are generated by yeasts as a result of the reaction between higher alcohols and acetyl co-A [36]. Higher alcohols are probably generated from the amino acid of pea proteins by the Ehrlich pathway [52].

As shown in Table 4, 2-phenylethyl acetate was produced in great quantity in the samples fermented with *Kluyveromyces* spp. It was shown that *K. marxianus* could produce this compound [53] and that *K. lactis* increased the amounts of this compound in the fermented soybean, okara [44].

In addition, hexyl acetate was identified in our fermented samples. This ester is not a common molecule found in beer. It is predicted that hexanol could be a direct precursor for this ester compound through the action of an alcohol acetyl transferase enzyme [53].

Other volatile compounds such as ethyl hexanoate and ethyl octanoate are produced by the lipid metabolism in yeasts and involve a reaction between ethanol and a fatty acid chain [48].

The presence of esters can add more complexity to pea products. In fact, esters can act in synergy with other molecules, thus influencing the final beer flavor at concentrations below the perception threshold [49].

To sum up, we can clearly see that most of the ester compounds were present at higher levels in co-cultures with *Kluyveromyces* sp. compared to *Torulaspora.* This highlights the fact that the different yeasts evaluated exert different metabolic activities. Moreover, the presence of these ester molecules might have generated a masking effect on the sensory defects in peas. Thus, further investigations are needed to identify the underlying phenomena that explain the modification of sensory perception in the presence of the following yeast species: *Kluyveromyces marxianus*, *Kluyveromyces lactis*, and *Torulaspora delbrueckii*.

## 4. Conclusions

A microbial co-culture of VEGE and yeasts modified the sensory perception of a pea protein-based product. The intensity of the leguminous and green perception was decreased compared to VEGE alone. Moreover, new descriptors were generated in the presence of yeasts. A hedonic evaluation should be carried out to evaluate the acceptability of the fermented products.

Analytical results have shown that most of the molecules responsible for the leguminous and green off-notes were degraded by VEGE and by VEGE and yeasts, which is not in agreement with the sensory results. Nevertheless, three molecules, (E)-2-heptenal, trans-2-methyl-2-butenal, and 6-methyl-5-hepten-2-one, were still found in the products with VEGE but absent in those with VEGE and yeasts. Thus, it will be necessary to increment the list of molecules potentially responsible for the off-notes and to determine their threshold perception in the pea matrix.

The presence of yeasts modified the sensory perception of a pea “yogurt-like” product. In particular, the beer/yeast descriptor, which is probably due to esters, could be responsible for the decrease of the leguminous and green off-notes. This potential masking effect should be further investigated.

In conclusion, the fermentation process using a co-culture of LAB and yeasts is of major industrial interest because this process can compensate for the sensory defects in a plant matrix without adding any artificial aroma compounds.

## Figures and Tables

**Table 1:** Fermentation characteristics of the different cultures.

	Bacteria Biomass (× 10^8^ CFU/mL)	Yeast Biomass (× 10^7^ CFU/mL)		Kinetic Parameters (g/L)			
	at tf	at t0	at tf *	tf (h)	Total Residual Sugar at tf	Lactic Acid at tf	Ethanol at tf
VEGE047	2.9 ± 0.2 ^b^	-	-	13.1 ± 0.5 ^ab^	25.4 ± 0.3 ^a^	3.5 ± 0.03 ^b^	-
VEGE047 + *K. marxianus*	4.2 ± 0.6 ^ab^	1.4 ± 0.1 ^b^	4.4 ± 0.5 ^a^	14.9 ± 0.9 ^a^	13.2 ± 0.1 ^d^	3.0 ± 0.05 ^d^	4.8 ± 0.007 ^a^
VEGE047 + *K. lactis*	6.0 ± 1.6 ^a^	3.4 ± 0.6 ^a^	4.1 ± 0.7 ^a^	13.1 ± 0.4 ^ab^	20.8 ± 0.6 ^b^	3.6 ± 0.03 ^a^	1.7 ± 0.004 ^c^
VEGE047 + *T. delbrueckii*	5.4 ± 0.3 ^a^	0.4 ± 0.1 ^b^	0.7 ± 0.1 ^a^	12.3 ± 0.01 ^b^	16 ± 0.3 ^c^	3.3 ± 0.02 ^c^	4.2 ± 0.02 ^b^

Each mean is based on three independent replicates. The values with letters of the same color were compared with each other. Values with the same letters are not significantly different (*p* > 0.05). * tf is the time needed to reach pH 4.55.

**Table 2:** Average intensity of sensory attributes determined for different fermented products using a scale ranging from 0 to 15.

	VEGE	VEGE *+ K. marxianus*	VEGE + *K. lactis*	VEGE + *T. delbrueckii*
Global intensity	4.660 b	7.033 a	6.390 a	6.900 a
Sour	2.257 b	5.000 a	4.250 a	5.400 a
Bitter	1.837 a	3.200 a	2.840 a	2.867 a
Astringent	3.730 a	4.733 a	4.243 a	4.933 a
Tangy	0.417 c	5.267 a	3.500 b	5.200 a
Sparkling	0.050 d	7.467 b	4.817 c	9.367 a
Green flavor/Vegetal	2.637 a	0.733 b	1.533 ab	0.867 b
Leguminous plant	3.723 a	1.147 b	1.500 b	0.800 b
Citrus fruit	0.400 a	0.300 a	0.850 a	0.200 a
Nut	0.267 a	0.167 a	0.333 a	0.200 a
Beer/yeast	0.183 c	5.000 a	3.150 b	2.800 b
Sourdough	0.243 b	1.260 ab	1.380 ab	1.800 a
Cultured apple cider	0.200 a	1.340 a	1.067 a	1.067 a

Mean values in the same row that are not followed by the same letter are significantly different (*p* < 0.05).

**Table 3:** Concentrations of the off-flavor molecules in the non-fermented and fermented samples (µg/L).

Volatile Compounds	Descriptors	Uninoculated	VEGE	VEGE + *K. marxianus*	VEGE + *K. lactis*	VEGE + *T. delbrueckii*	Detection Threshold ^a^
2-pentylfuran	Musty/earthy, mushroom, floral, buttery, rancid, green	304.3	46.4	56.7	148	55	6
Hexanal	Green, grass	181.3	<DL	<DL	<DL	<DL	4.5
2-ethylfuran	Beany, earthy, malty, sweet	77.7	28.1	29.7	56.7	32.8	
Butanal	Pungent, green, malty, chocolate, cocoa	54.9	<DL	<DL	<DL	<DL	
1-penten-3-ol	Green, vegetable, fruity	19.6	3.7	11	28.1	5.6	
(E)-2-octenal	Green, cucumber, musty/earthy, waxy, fatty, grass, banana, sweet	10.3	<DL	<DL	<DL	<DL	3
Nonanal	Aldehydic, fatty, green, geranium, floral, soapy, citrus, waxy	8.1	<DL	<DL	<DL	<DL	1
Heptanal	Green, fresh, fatty	5.7	<DL	<DL	<DL	<DL	3
2-nonanone	Green, earthy, grassy, fruity, sweet,	5.0	<DL	<DL	<DL	1.2	
2-methylpropanal	Aldehylic, grass, green, floral	4.0	0.7	40.9	70	39.6	6
1-octen-3-ol	Mushroom, earthy, burnt, green, vegetable, stale	3.9	2.2	2.9	6.7	2.9	1
(E)-2-heptenal	Pungent green, fatty	2.7	0.8	<DL	<DL	<DL	13
2-octanone	Green, floral, soapy, fruity, fatty	2.5	0.7	<DL	0.9	0.8	
Octanal	Aldehylic, green, soapy, citrus-like, sweet, waxy, fruity	2.5	<DL	<DL	<DL	<DL	0.7
(E)-2-Hexenal	Tea-like, green grass, almond, cherry, juicy, rancid	2.4	<DL	<DL	<DL	<DL	17
Decanal	Fresh, marine, aldehydic, iodized, soapy, grapefruit, bitter, sweet	1.04	<DL	<DL	<DL	<DL	0.1
6-methyl-5-hepten-2-one	Nutty, moldy, green, vegetable, citrus	0.5	0.5	<DL	<DL	<DL	2
Trans-2-methyl-2-butenal	Strong green-type odor and a fruity flavor	0.3	0.1	<DL	<DL	<DL	
1-hexanol	Green, musty/earthy, peanut hull, chemical-like, fruity, grassy	<DL	126.5	175.4	500.1	116.7	500
1-octanol	Moss, mushroom, green, vegetable, fatty, waxy, citrus, floral	<DL	<DL	4.4	<DL	2.9	

Concentrations are classified as per decreasing concentrations in the initial matrix. ^a^ Determined in water by orthonasal olfaction [38]. <DL: value inferior to the detection limit.

**Table 4:** Concentrations of esters in the fermented samples with yeasts (µg/L).

Esters Compounds	Odor Descriptor *	Threshold Level ^a^	Threshold Level in Beer ^b^	VEGE + *K. marxianus*	VEGE + *K. lactis*	VEGE + *T. delbrueckii*
Ethyl acetate	Ethereal, fruity, sweet, grape and rum-like	12,000	25,000	2095	3040	633
Propyl acetate	Solvent-like pungency, lifting, fusel, amyl alcohol, sweet and fruity	4700		9.2	39.1	>DL
Hexyl acetate	Green, fruity, sweet, fatty, fresh, apple and pear	670		73	5.7	>DL
Isobutyl acetate	Sweet, fruity, ethereal with an apple banana nuance	1600	500	14	6.5	>DL
2-methyl butyl acetate	Sweet, banana, fruity, ripe, estery and tropical with a juicy, fruit-like note			78	0.84	>DL
3-methyl butyl acetate	Sweet, banana, fruity with a ripe estery nuance	160	2000	40	4.9	6.8
2-phenylethyl acetate	Sweet, honey, floral rosy, with a slight yeasty honey note with a cocoa and balsamic nuance	1800	200	354	372	2.7
Ethyl propanoate	Sweet, ethereal, rummy, grape, winey and fermented with an eggnog nuance	1800		51	240	30
Ethyl isobutyrate	Citrus, fruity, sweet			45	16	1
Ethyl hexanoate	Sweet, fruity, pineapple, waxy, fatty and estery with a green banana nuance	80	200	5	>DL	10
Ethyl octanoate	Waxy, sweet, musty, pineapple and fruity with a creamy, dairy nuance	580	1000	2	>DL	3

^a^ Determined by orthonasal olfaction in 10% (*v/v*) ethanol solution adjusted to pH 3.5 with tartaric acid [45]. ^b^ Determined by orthonasal olfaction in beer [46]. * Descriptors presented in [47,48,49]

## Supplementary Materials

The following are available online at https://www.mdpi.com/2304-8158/9/3/349/s1. Table S1: Amount of residual sugars in the fermented samples (g/L), Table S2: Volatile compounds identified in the non-fermented and fermented pea samples (expressed as the surface area of the peaks).

## References

[1] Graça J., Godinho C.A., Truninger M. (2019). Reducing meat consumption and following plant-based diets: Current evidence and future directions to inform integrated transitions. Trends Food Sci. Technol..

[2] Silva A.R.A., Silva M.M.N., Ribeiro B.D. (2020). Health Issues and Technological Aspects of Plant-based Alternative Milk. Food Res. Int..

[3] McClements D.J., Newman E., McClements I.F. (2019). Plant-based Milks: A Review of the Science Underpinning Their Design, Fabrication, and Performance. Compr. Rev. Food Sci. Food Saf..

[4] Singh N. (2017). Pulses: An overview. J. Food Sci. Technol..

[5] Roland W.S., Pouvreau L., Curran J., van de Velde F., De Kok P. (2017). Flavor Aspects of Pulse Ingredients. Cereal Chem..

[6] Dahl W.J., Foster L.M., Tyler R.T. (2012). Review of the health benefits of peas (*Pisum sativum* L.). Br. J. Nutr..

[7] Iqbal A., Khalil I.A., Ateeq N., Sayyar Khan M. (2006). Nutritional quality of important food legumes. Food Chem..

[8] Lu Z.X., He J.F., Zhang Y.C., Bing D.J. (2019). Composition, physicochemical properties of pea protein and its application in functional foods. Crit. Rev. Food Sci. Nutr..

[9] Murat C., Bard M.-H., Dhalleine C., Cayot N. (2013). Characterisation of odour active compounds along extraction process from pea flour to pea protein extract. Food Res. Int..

[10] Schindler S., Zelena K., Krings U., Bez J., Eisner P., Berger R.G. (2012). Improvement of the Aroma of Pea (*Pisum sativum*) Protein Extracts by Lactic Acid Fermentation. Food Biotechnol..

[11] Bott L., Chambers E. (2006). Sensory Characteristics of Combinations of Chemicals Potentially Associated with Beany Aroma in Foods. J. Sens. Stud..

[12] Tangyu M., Muller J., Bolten C.J., Wittmann C. (2019). Fermentation of plant-based milk alternatives for improved flavour and nutritional value. Appl. Microbiol. Biotechnol..

[13] Blandino A., Al-Aseeri M.E., Pandiella S.S., Cantero D., Webb C. (2003). Cereal-based fermented foods and beverages. Food Res. Int..

[14] Spitaels F., Wieme A.D., Janssens M., Aerts M., Daniel H.-M., Van Landschoot A., De Vuyst L., Vandamme P. (2014). The Microbial Diversity of Traditional Spontaneously Fermented Lambic Beer. PLoS ONE.

[15] Aidoo K.E., Nout M.J.R., Sarkar P.K. (2006). Occurrence and function of yeasts in Asian indigenous fermented foods. FEMS Yeast Res..

[16] Park Y.K., Lee J.H., Mah J.-H. (2019). Occurrence and reduction of biogenic amines in traditional Asian fermented soybean foods: A review. Food Chem..

[17] Onda T., Yanagida F., Uchimura T., Tsuji M., Ogino S., Shinohara T., Yokotsuka K. (2003). Analysis of Lactic Acid Bacterial Flora during Miso Fermentation. Food Sci. Technol. Res..

[18] Feng X.M., Ostenfeld Larsen T., Schnürer J. (2007). Production of volatile compounds by Rhizopus oligosporus during soybean and barley tempeh fermentation. Int. J. Food Microbiol..

[19] Xu D., Wang P., Zhang X., Zhang J., Sun Y., Gao L., Wang W. (2020). High-throughput sequencing approach to characterize dynamic changes of the fungal and bacterial communities during the production of sufu, a traditional Chinese fermented soybean food. Food Microbiol..

[20] Koutsoumanis K., Allende A., Alvarez-Ordóñez A., Bolton D., Bover-Cid S., Chemaly M., Davies R., Cesare A.D., Hilbert F., Lindqvist R. (2019). Update of the list of QPS-recommended biological agents intentionally added to food or feed as notified to EFSA 10: Suitability of taxonomic units notified to EFSA until March 2019. EFSA J..

[21] Sieuwerts S., de Bok F.A.M., Hugenholtz J., van Hylckama Vlieg J.E.T. (2008). Unraveling Microbial Interactions in Food Fermentations: From Classical to Genomics Approaches. Appl. Environ. Microbiol..

[22] Schindler S., Wittig M., Zelena K., Krings U., Bez J., Eisner P., Berger R.G. (2011). Lactic fermentation to improve the aroma of protein extracts of sweet lupin (*Lupinus angustifolius*). Food Chem..

[23] Yousseef M., Lafarge C., Valentin D., Lubbers S., Husson F. (2016). Fermentation of cow milk and/or pea milk mixtures by different starter cultures: Physico-chemical and sensorial properties. LWT Food Sci. Technol..

[24] Ben-Harb S., Saint-Eve A., Panouillé M., Souchon I., Bonnarme P., Dugat-Bony E., Irlinger F. (2019). Design of microbial consortia for the fermentation of pea-protein-enriched emulsions. Int. J. Food Microbiol..

[25] Chiba H., Takahashi N., Sasaki R. (1979). Enzymatic Improvement of Food Flavor II. Removal of Beany Flavor from Soybean Products by Aldehyde Dehydrogenase. Agric. Biol. Chem..

[26] Hirst M.B., Richter C.L. (2016). Review of Aroma Formation through Metabolic Pathways of Saccharomyces cerevisiae in Beverage Fermentations. Am. J. Enol. Vitic..

[27] Picque D., Corrieu G. (1992). Characterization and classification of lactic acid bacteria based on their acidification kinetics. Food Sci. Technol. Lebensm. Wiss. Technol..

[28] Le Boucher C., Courant F., Jeanson S., Chereau S., Maillard M.-B., Royer A.-L., Thierry A., Dervilly-Pinel G., Le Bizec B., Lortal S. (2013). First mass spectrometry metabolic fingerprinting of bacterial metabolism in a model cheese. Food Chem..

[29] Merico A., Galafassi S., PiÅ¡kur J., Compagno C. (2009). The oxygen level determines the fermentation pattern in *Kluyveromyces lactis*. FEMS Yeast Res..

[30] Lane M.M., Morrissey J.P. (2010). *Kluyveromyces marxianus:* A yeast emerging from its sister’s shadow. Fungal Biol. Rev..

[31] Hu Y., Piao C., Chen Y., Zhou Y., Wang D., Yu H., Xu B. (2019). Soybean residue (okara) fermentation with the yeast *Kluyveromyces marxianus*. Food Biosci..

[32] McMahon K.M., Culver C., Castura J.C., Ross C.F. (2017). Perception of carbonation in sparkling wines using descriptive analysis (DA) and temporal check-all-that-apply (TCATA). Food Qual. Prefer..

[33] Fonseca G.G., Heinzle E., Wittmann C., Gombert A.K. (2008). The yeast Kluyveromyces marxianus and its biotechnological potential. Appl. Microbiol. Biotechnol..

[34] Lederer C.L., Bodyfelt F.W., McDaniel M.R. (1991). The Effect of Carbonation Level on the Sensory Properties of Flavored Milk Beverages. J. Dairy Sci..

[35] Verstrepen K.J., Derdelinckx G., Dufour J.-P., Winderickx J., Thevelein J.M., Pretorius I.S., Delvaux F.R. (2003). Flavor-active esters: Adding fruitiness to beer. J. Biosci. Bioeng..

[36] Azarnia S., Boye J.I., Warkentin T., Malcolmson L. (2011). Changes in volatile flavour compounds in field pea cultivars as affected by storage conditions. Int. J. Food Sci. Technol..

[37] Murat Chloe Gourrat K., Jerosch H., Cayot N. (2012). Analytical comparison and sensory representativity of SAFE, SPME, and Purge and Trap extracts of volatile compounds from pea flour. Food Chem..

[38] Buttery R.G., Orts W.J., Takeoka G.R., Nam Y. (1999). Volatile Flavor Components of Rice Cakes. J. Agric. Food Chem..

[39] Heng L., Vincken J.-P., Koningsveld G van Legger A., Gruppen H., Boekel T van Roozen J., Voragen F. (2006). Bitterness of saponins and their content in dry peas. J. Sci. Food Agric..

[40] Achouri A., Boye J., Zamani Y. (2006). Identification of volatile compounds in soymilk using solid-phase microextraction-gas chromatography. Food Chem..

[41] Blagden T.D., Gilliland S.E. (2005). Reduction of Levels of Volatile Components Associated with the “Beany” Flavor in Soymilk by Lactobacilli and Streptococci. J. Food Sci..

[42] Ben-Harb S., Irlinger F., Saint-Eve A., Panouillé M., Souchon I., Bonnarme P. (2020). Versatility of microbial consortia and sensory properties induced by the composition of different milk and pea protein-based gels. LWT.

[43] Smit B.A., Engels W.J.M., Smit G. (2009). Branched chain aldehydes: Production and breakdown pathways and relevance for flavour in foods. Appl. Microbiol. Biotechnol..

[44] Vong W.C., Liu S.-Q. (2018). Bioconversion of green volatiles in okara (soybean residue) into esters by coupling enzyme catalysis and yeast (Lindnera saturnus) fermentation. Appl. Microbiol. Biotechnol..

[45] Peinado R.A., Moreno J., Bueno J.E., Moreno J.A., Mauricio J.C. (2004). Comparative study of aromatic compounds in two young white wines subjected to pre-fermentative cryomaceration. Food Chem..

[46] Engan S. (1972). Organoleptic threshold values of some alcohols and esters in beer. J. Inst. Brew..

[47] Dennis E.G., Keyzers R.A., Kalua C.M., Maffei S.M., Nicholson E.L., Boss P.K. (2012). Grape Contribution to Wine Aroma: Production of Hexyl Acetate, Octyl Acetate, and Benzyl Acetate during Yeast Fermentation Is Dependent upon Precursors in the Must. J. Agric. Food Chem..

[48] Pires E.J., Teixeira J.A., Brányik T., Vicente A.A. (2014). Yeast: The soul of beer’s aroma—A review of flavour-active esters and higher alcohols produced by the brewing yeast. Appl. Microbiol. Biotechnol..

[49] Meilgaard M.C., Dalgliesh C.E., Clapperton J.F. (1979). Beer flavour terminology^1^. J. Inst. Brew..

[50] Plata C., Millán C., Mauricio J.C., Ortega J.M. (2003). Formation of ethyl acetate and isoamyl acetate by various species of wine yeasts. Food Microbiol..

[51] Lyumugabe F., Bajyana Songa E., Wathelet J.P., Thonart P. (2013). Volatile compounds of the traditional sorghum beers “ikigage” brewed with Vernonia amygdalina “umubirizi”. Cerevisia.

[52] Hazelwood L.A., Daran J.-M., van Maris A.J.A., Pronk J.T., Dickinson J.R. (2008). The Ehrlich Pathway for Fusel Alcohol Production: A Century of Research on Saccharomyces cerevisiae Metabolism. Appl. Environ. Microbiol..

[53] Liu S.-Q., Holland R., Crow V.L. (2004). Esters and their biosynthesis in fermented dairy products: A review. Int. Dairy J..

