# Supplementary material for: Sensory Improvement of a Pea Protein-Based Product Using Microbial Co-Cultures of Lactic Acid Bacteria and Yeasts
Source: Foods. 2020 Mar 17;9(3):349. doi: 10.3390/foods9030349 (PMC7143830; doi:10.3390/foods9030349)
Supplement: Supplementary file 1 [file foods-09-00349-s001.pdf]

Table S 1 Amount of residual sugars in the fermented samples (g/L).

	Residual sugars at tf		
	**Sucrose**	**Glucose**	**Fructose**
**VEGE**	24.6 a	< 0.001 d	0.8 c
**VEGE + KM**	< 0.001 b	3.3 c	9.9 b
**VEGE + KL**	0.27 b	7.5 a	13 a
**VEGE + TOR**	0.4 b	5.6 b	10 b

Means in the same column with different letters are significantly different ($p < 0.05$).

Table S 2 Volatile compounds identified in the non-fermented and fermented pea samples (expressed as the surface area of the peaks).

Families	Volatile compounds	Surface area				
		Uninoculated	VEGE	VEGE + KM	VEGE + KL	VEGE +TOR
Aldehydes	2-methylpropanal	2.29E+06	3.99E+05	2.3E+07	4.01E+07	2.25E+07
	Butanal	8.46E+06	< DL	< DL	< DL	< DL
	3-methylbutanal	5.08E+06	< DL	3.3E+06	8.65E+06	2.88E+06
	2-methylbutanal	2.99E+06	< DL	2.7E+06	1.07E+07	3.82E+06
	Pentanal	9.48E+06	< DL	< DL	< DL	< DL
	Trans -2-methyl-2-butenal	1.27E+05	5.11E+04	< DL	< DL	< DL
	Hexanal	6.85E+07	< DL	< DL	< DL	< DL
	2-ethyl-2-butenal	5.42E+04	3.19E+04	< DL	< DL	< DL
	(E)-2-hexenal	5.81E+04	< DL	< DL	< DL	< DL
	Heptanal	2.59E+06	< DL	< DL	< DL	< DL
	(E)-2-heptenal	6.35E+04	2.01E+04	< DL	< DL	< DL
	Benzaldehyde	< DL	1.19E+05	< DL	< DL	< DL
	Octanal	2.44E+06	< DL	< DL	< DL	< DL
	2-ethyl-2-hexenal	3.25E+05	1.65E+05	< DL	< DL	< DL
	Nonanal	2.06E+06	< DL	< DL	< DL	< DL
	3-ethyl benzaldehyde	5.96E+04	8.25E+03	< DL	< DL	< DL
	Decanal	2.15E+05	< DL	< DL	< DL	< DL
	Cis-undec-4-enal	< DL	3.66E+04	< DL	< DL	< DL
	(E)-2-octenal	3.71E+05	< DL	< DL	< DL	< DL
	Dodecanal	9.16E+04	< DL	< DL	< DL	< DL
Ketones	Acetone	4.24E+06	< DL	< DL	1.14E+06	8.93E+04
	2,3-butanedione	5.93E+04	4.18E+06	< DL	6.48E+06	< DL
	2-butanone	< DL	5.76E+05	< DL	3.14E+06	6.81E+05
	2-pentanone	5.93E+05	1.43E+05	< DL	1.63E+06	4.29E+05
	2,3-pentanedione	< DL	4.62E+06	< DL	3.49E+06	1.99E+05
	4-methyl-2-pyrazolin-5-one	< DL	5.75E+04	< DL	< DL	< DL
	2,3-Hexanedione	< DL	5.79E+04	< DL	< DL	< DL
	2-hexanone	5.90E+05	1.87E+05	< DL	8.60E+05	1.82E+05
	4-methyl-3-penten-2-one	< DL	< DL	< DL	1.02E+06	< DL
	1-(2-furanyl)-1-propanone	3.20E+05	3.09E+04	< DL	< DL	6.01E+04
	2- heptanone	1.37E+07	3.91E+06	8.2E+05	1.38E+07	3.58E+06
	3-ethylcyclopentanone	1.15E+05	3.70E+04	< DL	< DL	6.61E+04
	(Z)-6-octen-2-one	< DL	< DL	1.8E+06	7.59E+06	2.16E+06
	6-methyl-5-hepten-2-one	1.1E+05	1.04E+05	< DL	< DL	< DL
	2-octanone	1.12E+06	3.12E+05	< DL	1.56E+06	3.77E+05
	Acetophenone	1.82E+05	< DL	< DL	< DL	< DL
	2-nonanone	2.35E+06	5.22E+05	< DL	< DL	5.57E+05
	(E, E) -3,5-octadien-2-one	2.22E+06	7.82E+04	< DL	1.57E+06	3.07E+05

	3-nonen-2-one	< DL	9.03E+03	< DL	< DL	< DL
	2-decanone	< DL	< DL	< DL	1.63E+06	< DL
	6-undecanone	1.14E+05	< DL	< DL	< DL	< DL
	Ethanol	< DL	< DL	5.4E+07	6.50E+07	4.78E+07
	1-propanol	< DL	1.12E+05	< DL	6.90E+06	4.28E+06
	2-methyl-1-propanol	< DL	< DL	2.7E+08	1.56E+08	4.58E+07
	1-butanol	< DL	2.38E+05	< DL	9.77E+05	3.77E+05
	1-penten-3-ol	8.85E+05	1.67E+05	5.0E+05	1.27E+06	2.55E+05
	3-methyl-1-butanol	< DL	< DL	4.4E+08	1.34E+08	1.13E+08
	2-methyl-1-butanol	< DL	< DL	5.7E+07	5.57E+07	2.90E+07
**Alcohols**	1-pentanol	1.51E+06	1.48E+06	1.6E+06	5,34E+06	1.10E+06
	2,3-butanediol	< DL	< DL	2.4E+06	< DL	3.73E+05
	1-hexanol	< DL	7.30E+06	1.0E+07	2.89E+07	6.74E+06
	(S)-2-heptanol	< DL	< DL	1.0E+06	< DL	2.53E+05
	4-ethyl phenol	7.88E+05	2.43E+05	3.0E+05	5.95E+05	3.04E+05
	1-Heptanol	< DL	3.12E+05	< DL	< DL	4.01E+05
	1-octen-3-ol	4.86E+05	2.79E+05	3.6E+05	8.45E+05	3.71E+05
	1-adamantanol	6.57E+05	< DL	< DL	< DL	1.01E+05
	1-octanol	< DL	< DL	**3.9E+05**	< DL	2.59E+05
	Phenylethyl alcohol	< DL	< DL	< DL	< DL	1.70E+06
	3-methyl furan	5.62E+05	3.10E+05	< DL	4.99E+05	2.21E+05
	2-ethyl furan	1.98E+07	7.15E+06	7.5E+06	1.44E+07	8.35E+06
	2,4-dimethylfuran	< DL	< DL	< DL	1.18E+07	< DL
	2-propyl furan	5.62E+05	1.49E+05	3.7E+05	3.71E+05	1.47E+05
	Furfural	6.44E+04	< DL	< DL	< DL	< DL
**Furans**	2-n- butyl furan	8.84E+04	**3.36E+05**	3.5E+05	< DL	3.93E+05
	2-pentyl furan	7.94E+07	1.21E+07	1.5E+07	3.86E+07	1.44E+07
	2-hexyl furan	1.85E+05	1.60E+04	< DL	< DL	< DL
	2-n-heptyl furan	1.58E+05	< DL	< DL	< DL	< DL
	2-n-octylfuran	5.62E+04	< DL	< DL	< DL	< DL
	Trans-2-(2-pentenyl) furan	1.52E+06	3.50E+05	4.0E+05	7.80E+05	4.29E+05
	Acetic acid, ethenyl ester	< DL	< DL	5.9E+05	< DL	1.83E+06
	Butyl isocyanatoacetate	< DL	< DL	1.4E+06	< DL	8.38E+05
	Acetic acid, methyl ester	< DL	< DL	< DL	5.09E+05	< DL
	Ethyl acetate	< DL	< DL	5.4E+08	5.53E+08	1.13E+08
	Propanoic acid, ethyl ester	< DL	< DL	1.4E+07	6.44E+07	7.94E+06
**Esters**	n-propyl acetate	< DL	< DL	3.09E+06	1.31E+07	< DL

Propanoic acid, 2-methyl-, ethyl ester	< DL	< DL	2.5E+07	8.66E+05	6.07E+04
Isobutyl acetate	< DL	< DL	6.9E+06	3.21E+06	< DL
Acetic acid, butyl ester	< DL	< DL	< DL	4.04E+05	< DL
1 butanol, 3-methyl-, acetate	< DL	< DL	1.9E+07	2.36E+06	3.21E+05
1 butanol, 2-methyl-, acetate	< DL	< DL	4.6E+06	4.96E+05	< DL
Acetic acid, pentyl ester	< DL	< DL	5.0E+05	1.31E+06	< DL
1-butanol, 3-methyl-, propanoate	< DL	< DL	9.4E+05	< DL	< DL
Hexanoic acid, ethyl ester	< DL	< DL	1.1E+06	< DL	5.61E+05
Propanoic acid, 2-methyl, 3-methylbutyl ester	< DL	< DL	4.3E+05	< DL	< DL
Heptanoic acid, ethyl ester	< DL	< DL	< DL	< DL	7.00E+04
Acetic acid, hexyl ester	< DL	< DL	5.1E+06	2.69E+06	< DL
Octanoic acid, ethyl ester	< DL	< DL	3.1E+05	< DL	4.2E+05
Acetic acid, 2-phenylethyl ester	< DL	< DL	9.2E+06	1.44E+07	1.34E+05
Propanoic acid, 2-phenylethyl ester	< DL	< DL	3.9E+05	< DL	< DL
Propanoic acid, 2-methyl-, 2-phenylethyl ester	< DL	< DL	3.5E+05	< DL	< DL
Decanoic acid, ethyl ester	< DL	< DL	< DL	< DL	6.94E+04
